# Cytoskeletal remodeling via CAMSAP3 downregulation drives resistance to osimertinib in NSCLC cells

**DOI:** 10.1038/s41419-025-08299-0

**Published:** 2025-12-11

**Authors:** Fei Yang, Zhen Wang, Xiao Han, Jinjin Zhong, Zhanwu Hou, Shuying Bian, Jiangang Long, Huadong Liu

**Affiliations:** 1 Center for Mitochondrial Biology and Medicine, The Key Laboratory of Biomedical Information Engineering of Ministry of Education, School of Life Science, Xi’an Jiaotong University, Xi’an, China; 2 School of Life Sciences and Health, University of Health and Rehabilitation Sciences, Qingdao, China; 3 Department of Pathogen Biology, School of Basic Medical Science, Xi’an Medical University, Xi’an, China

**Keywords:** Non-small-cell lung cancer, Cytoskeleton

## Abstract

Osimertinib, also known as AZD9291, is a highly potent and selective EGFR mutants (including exon 19 deletion, L858R/T790M) inhibitor that significantly inhibits EGFR phosphorylation signaling. However, acquired resistance to osimertinib is inevitable in the treatment of non-small cell lung cancer (NSCLC). Microtubules, key cytoskeletal components involved in intracellular cargo transport, mediate EGFR-endosomal recycling, yet their specific role in osimertinib resistance remains to be elucidated. In this study, we found that centrosomal microtubule formation was increased in osimertinib-resistant NSCLC cells, and calmodulin-regulated spectrin-associated protein 3 (CAMSAP3) was identified as the key molecule responsible for the change of microtubule morphology. Genetic modulation via CAMSAP3 silencing in both osimertinib-sensitive cells (in vitro) and xenograft models (in vivo) enhanced microtubule clustering and resistance to osimertinib, whereas CAMSAP3 overexpression in resistant cells partially restored microtubule organization and drug sensitivity. Furthermore, we demonstrated that full-length CAMSAP3 is essential for proper localization of the microtubule-dependent endosomal-lysosomal system. CAMSAP3 depletion caused EGFR translocation to the perinuclear microtubule organizing center (MTOC), thereby blocking plasma membrane recycling and promoting lysosomal degradation. These findings establish CAMSAP3 as a key regulator of EGFR signaling and osimertinib response in NSCLC, suggesting its therapeutic potential for overcoming drug resistance in lung cancer.

## Introduction

First-generation EGFR tyrosine kinase inhibitors (TKIs), such as gefitinib and erlotinib, and second-generation TKIs including afatinib and dacomitinib have consistently demonstrated high efficacy in treating metastatic non-small cell lung cancer with specific EGFR mutations, particularly exon 19 deletions and the L858R mutation [1, 2]. However, drug resistance develops rapidly, with the gatekeeper T790M mutation of the EGFR gene leading to drug resistance to first- and second-generation TKIs [3–5]. Osimertinib, a third-generation EGFR TKI that irreversibly inhibits EGFR-T790M mutations, is the preferred therapeutic option for EGFR-mutant NSCLC [6, 7]. However, resistance to osimertinib is also inevitable. The mechanisms of acquired resistance to osimertinib are highly heterogeneous, such as T790M deletion, C797S mutation, MET and PTK7 amplification [8–10]. However, the mechanism of osimertinib resistance remains unknown in ~40% of patients.

Wild-type EGFR is degraded within a short period of time after activation by ligands such as EGF by endocytosis, a cellular process that selectively internalizes cell surface proteins through plasma membrane invagination into endosomal vesicles for degradation [11, 12]. The EGFR-T790M mutant has a propensity to heterodimerize with ErbB2 and exhibits defects in the endosome-lysosome pathway. It escapes CBL-mediated ubiquitination and subsequent lysosomal degradation, resulting in activated, phosphorylated EGFR accumulating in endosomes or recycling to the cell surface. This process amplifies downstream signaling and survival pathways [13]. Recycling endosomes are transported through microtubules, along with other organelles such as the Golgi apparatus and lysosomes; this process facilitates cellular processes including tumor cell migration and invasion [14, 15]. Microtubule-regulated EGFR trafficking has been reported to be associated with TKI resistance [16]. Microtubule detyrosination promotes kinesin family member 3C (KIF3C)-mediated enhancement of endosomal recycling of EGFR, leading to prolonged activation of PI3K/Akt/mTOR signaling [17]. Previous studies have shown that the faster recycling of EGFR back to the cell surface activates the downstream ERK1/2 pathway and increases sensitivity to EGFR inhibitors [18].

The formation of centrosomal microtubules is contingent upon the initiating formation of microtubules (called “nucleation”) and the fixation of the minus end of microtubules at the MTOC (called “anchoring”) [19–21]. However, non-centrosomal microtubules predominate in epithelial cells. Release of microtubules (MTs) from centrosomes post-nucleation is pivotal for epithelial MT organization [22–24]. Several microtubule-binding proteins, particularly CAMSAP3, have been reported to regulate microtubule nucleation and anchoring in epithelial cells. CAMSAP3 knockdown in NSCLC lines (H460, A549, H23) has been shown to increase the levels of hypoxia-inducible factor-1β (HIF-1β) and its downstream targets vascular endothelial growth factor A (VEGFA), matrix metalloproteinases MMP2 and MMP9, thereby promoting NSCLC invasiveness [25].

In this study, we identified CAMSAP3 as a pivotal regulator of microtubule morphology in osimertinib-resistant cells. The depletion of CAMSAP3 altered osimertinib sensitivity of H1975 or HCC827 cell lines. Further studies revealed that microtubule morphology as well as the endosomal-lysosomal system exhibited similar localization in drug-resistant cells and CAMSAP3 knockdown cells, suggesting that CAMSAP3 further affects the endosomal-lysosomal system through microtubule network remodeling. In conclusion, our findings establish CAMSAP3 as a key regulator of EGFR signaling and osimertinib response in NSCLC, highlighting its therapeutic potential for overcoming drug resistance in lung cancer.

## Materials and methods

### Osimertinib-resistant H1975/HCC827 cell lines

The H1975 and HCC827 NSCLC cell lines were purchased from the National Collection of Authenticated Cell Cultures and authenticated by short tandem repeat (STR) profiling. RPMI 1640 medium supplemented with 10% fetal bovine serum, 0.1 mg/mL streptomycin and 100 U/mL penicillin was used to culture H1975/HCC827 cells. To establish drug-resistant cells, H1975/HCC827 cells were cultured in RPMI 1640 medium containing different concentrations of osimertinib for 4 months. Resistance was confirmed when cells proliferated normally in medium containing 1 μmol/L osimertinib. All cell lines were subjected to regular testing for mycoplasma contamination and were subsequently verified to be mycoplasma-negative.

### Immunofluorescent staining

For immunofluorescent staining experiments, cells were fixed in 100% ice-cold methanol for 5 min at −20 °C, then washed three times with PBS, and blocked with 5% bovine serum albumin (BSA, Solarbio, Beijing, China) at room temperature for 1 h. Cells were incubated with primary antibodies for 3 h and secondary antibodies for 1 h. Images of the stained cells were captured using a Super-resolution Confocal Microscope (Leica TCS SP8 STED 3X). ImageJ was employed to analyze fluorescence colocalization.

### MTT assay

Cell viability was assessed by MTT assay. After treatment, the supernatant of cell medium was discarded, and 100 μL of 1 mg/mL MTT solution within serum-free medium was added into each well and incubated at 37 °C for 3 h. After that, the supernatant with MTT was discarded, followed by the addition of 100 μL of dimethyl sulfoxide (DMSO, Solarbio, Beijing, China). The absorbance at 490 nm was measured by a microplate reader.

### Crystal violet staining assay

1 × 10^3^ cells were seeded in a 12-well plate and cultured for 2 days. After treatment, cells were fixed by 4% paraformaldehyde for 7 min and stained by 0.1% crystal violet for 15 min, The picture was obtained using light microscope. After that, 100 μL of 10% acetic acid was used to dissolve crystal violet to measure cell survival. The absorbance at 570 nm was measured with a microplate reader.

### Quantitative real‑time PCR (qRT‑PCR)

After three washes with PBS, mRNA was extracted by TRIzol Reagent (Invitrogen). 1 μg of RNA was used for cDNA reverse transcription with 5X RT Premix (AG11706, Accurate Biology). SYBR Green Premix Pro (AG11701, Accurate Biology) was used to amplify genes in ABI 7500 (Applied Biosystems). β-actin was taken as control. The qPCR primers are shown in Table S1 (in Supplementary Materials).

### Western blot analysis

After PBS washing, cells were harvested in IP lysis buffer (P0013, Beyotime Biotechnology), supplemented with PMSF and phosphatase inhibitors. Then the cell lysate was collected into 1.5 mL tubes, followed by centrifugation at 4 °C for 10 min at 12,000 rpm speed. Equal amounts of protein were separated on SDS-PAGE gels and transferred to NC membranes (Millipore). The membranes were blocked with 5% milk in TBST (20 mM Tris-HCl, 150 mM NaCl, 0.1% Tween-20, pH 7.6) at room temperature for 1 h. After TBST washing, primary antibodies were added and incubated at 4 °C overnight, and the secondary antibody was incubated for 1 h at room temperature. ECL kit (Pierce) was used to visualize by a chemiluminescent imaging system. Antibodies used are as indicated: CAMSAP3, SAB4200415, Sigma-Aldrich; Tubulin beta, M/R20005, Abmart; Tubulin gamma, T55405s, Abmart; Phospho-EGFR (Y1068) 3777T, Cell Signaling Technology; EGFR, sc-373746, Santa Cruz Biotechnology; Phospho-ERK1/2 (T202/Y204), TA1015, Abmart; ERK1/2, T40071, Abmart; EEA1, 3288T, Cell Signaling Technology; RAB11A, D4F5, Cell Signaling Technology; GAPDH, AC002, ABclonal; LAMP1, 9091, Cell Signaling Technology.

### siRNA transfection

Gene-specific siRNAs were purchased from Shanghai ShengGong (Shanghai, China). Cells were transfected with two independent siRNAs by using INTERFERin® (Polyplus-transfection S.A., Illkirch, France) according to the manufacturer’s protocols. siRNA sequences for knocking down target genes are listed in Table S2 (in Supplementary Materials).

### Lentiviral shRNA constructs and infection

Lentiviral shRNA against CAMSAP3 and scramble control shRNA were constructed according to previous report [10]. For lentiviral production, 293T cells were co-transfected using PEI as the transfection reagent with the shRNA vector (the gene of interest plasmid) along with packaging plasmids psPAX2 (Addgene 12260) and pMD2.G (Addgene 12259) at a ratio of 1:1.5:2 (shRNA vector: psPAX2: pMD2.G) and a ratio of 1:6 (PEI to total DNA). Viral supernatant was harvested 48 h post-transfection, filtered (0.45 μm), and immediately used for infection. To determine optimal infection conditions, we performed preliminary titration experiments indicating that this viral concentration achieved >60% infection efficiency (validated by parallel GFP-expressing lentiviral control). H1975 cells were infected with a 1:1 mixture of viral supernatant and fresh culture medium (final volume ratio) at ~60–70% confluence, 8 μg/mL polybrene was added into medium at the same time and incubated for 48 h, then the medium was supplemented with 2 μg/mL puromycin for screening. The sequences of shRNA used to knock down the indicated genes are listed in Table S3 (in Supplementary Materials).

### Animal experiments

To evaluate the impact of CAMSAP3 knockdown on osimertinib-resistant NSCLC, twenty 5-week-old female nude mice were obtained from GemPharmatech Laboratory Animal Co. Ltd (Chengdu, China) for xenograft tumor generation. The mice were randomly assigned to four groups: shCtrl_Ctrl, shCtrl_Osimertinib, shCAMSAP3_Ctrl, and shCAMSAP3_Osimertinib. Once the maximum tumor size reached ~100 mm^3^, the mice were administered either saline (vehicle control) or osimertinib (5 mg/kg body weight, once per day, oral gavage). Separately, to evaluate whether the combination of chloroquine (CQ) and osimertinib inhibits the growth of osimertinib-resistant cells, twenty 5-week-old female nude mice were obtained from the same vendor to establish H1975OR xenograft tumor models. Once the tumor volume reached ~50 mm³, the mice were randomly assigned to four groups (n = 5 per group): H1975OR_Ctrl, H1975OR_Osimertinib, H1975OR_CQ, and H1975OR_Osimertinib+CQ. The mice then received daily oral gavage of the following: saline (vehicle control), osimertinib (5 mg/kg body weight), CQ (75 mg/kg body weight), or a combination of osimertinib and CQ (at the same doses). At predetermined time points, the length (L) and width (W) of the tumors were measured. The tumor volume was calculated using the formula: (L × W^2^)/2. Tumors were harvested at the conclusion of the final measurement for the purpose of further analysis. No statistical methods were used to determine the sample size. The investigators were blind to group allocation during measurement. All animal protocols were approved by the Animal Care and Use Committee of Xi’an Jiaotong University’s School of Life Science and Technology, following the university’s established guidelines.

### Plasmids transfection

For transfection, 1 μg of plasmid was added in 200 μL opti-MEM, and then 5 μL PEI transfection reagent was added. The mix was added into the medium without any antibiotic after 15 min of incubation. After 4–6 h, the medium was replaced by fresh complete medium. 48–72 h later, cells were harvested for analysis. The sequences of the constructed plasmids are shown in Table S4 (in Supplementary Materials).

### Lysosomal pH assay

LysoSensor Green DND-189 (40767ES50, Yeason, China) was used to detect lysosomal pH. Cells were stained with 1 μM Lysotracker Green DND-189 diluted in RPMI-1640 at room temperature for 10 min, washed twice with PBS and added fresh medium. Hoechst 33342 (HY-15559, MCE, China) was used to stain nucleus. Images of the marked cells were captured using a Super-resolution Confocal Microscope (Leica TCS SP8 STED 3X) and the fluorescence intensity was detected and analyzed using ImageJ software.

### Statistical analysis

Excel was used for statistical analysis. The between-group variance was analyzed by unpaired two-tailed Student’s t test. Data represent as the mean ± SD from at least three independent experiments. There were statistically significant differences between two groups when *P* < 0.05.

## Results

### Microtubule remodeling accompanies osimertinib resistance in NSCLC cells

**Fig. 1:**
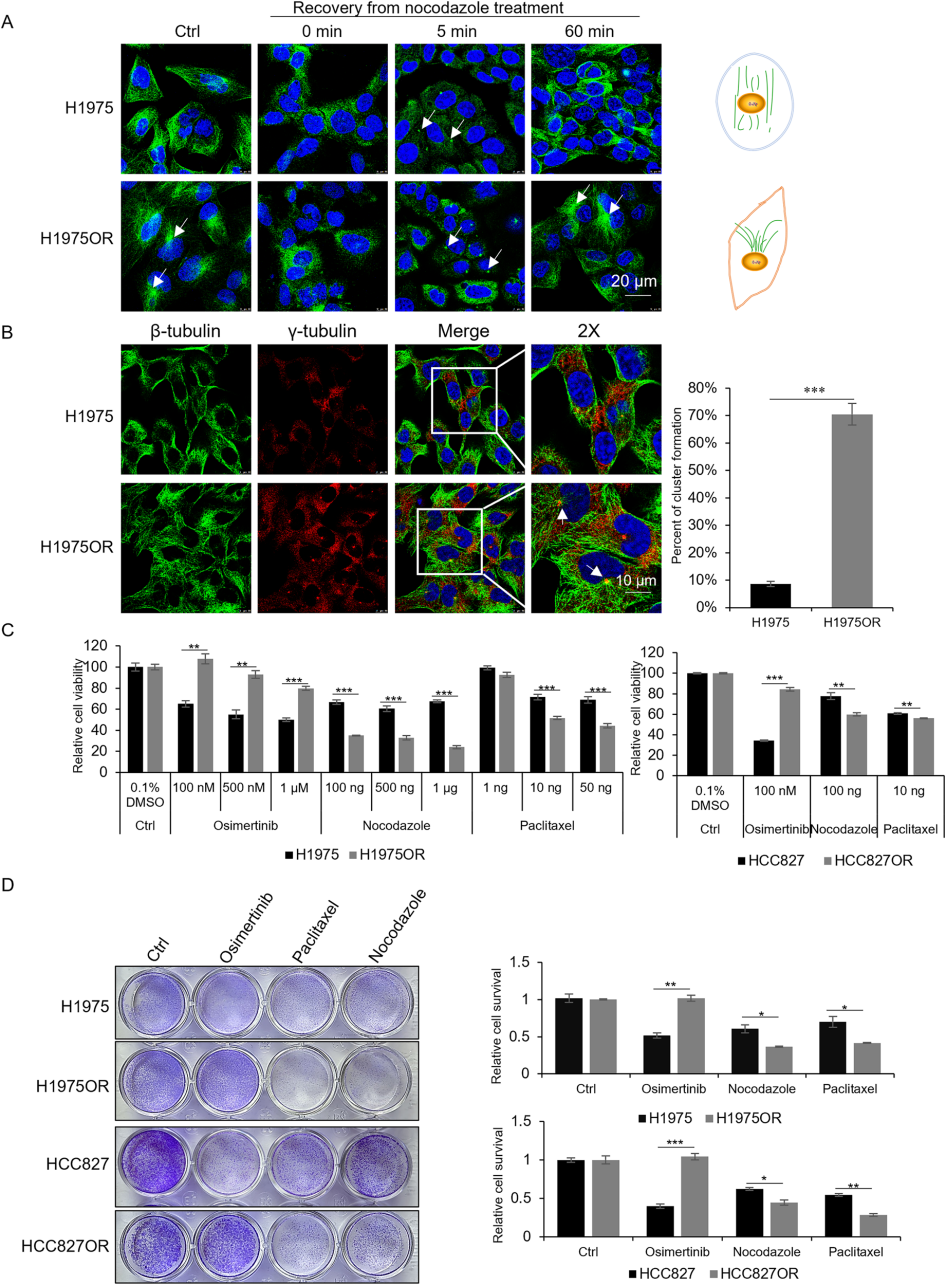
Microtubule dynamics are altered in osimertinib-resistant cells. **A** Immunofluorescence staining for β-tubulin in H1975/H1975OR cells treated with nocodazole (1 μg/mL) for 1 h and washout after 0, 5, 60 min, respectively. The arrowheads indicate nucleation positions. DAPI stains nuclei (Scale bars, 20 μm). **B** Co-immunofluorescence staining for β-tubulin and γ-tubulin in H1975/H1975OR cells. Centrosomes were stained with anti-γ-tubulin antibodies. The arrowheads indicate the positions of centrosomes. DAPI stains nuclei (Scale bars, 10 μm). The number of cells with a cluster of microtubules in (**B**) was quantified, in which 100 cells were analyzed per experiment, ***P < 0.001. **C** The cell viability of H1975/H1975OR and HCC827/HCC827OR cells after being treated with osimertinib, nocodazole and paclitaxel for 48 h was measured by MTT assays (mean ± SD, n = 6), **P < 0.01, ***P < 0.001. **D** Inhibition of cell survival in H1975/H1975OR and HCC827/HCC827OR cells by osimertinib (1 μM for H1975/H1975OR; 100 nM for HCC827/HCC827OR), nocodazole (100 ng/mL) and paclitaxel (10 ng/mL) for 48 h was measured by Crystal violet staining assay (mean ± SD, n = 3), *P < 0.05, **P < 0.01, ***P < 0.001.

Microtubules and their post-translational modifications have been implicated in EGFR transport and chemotherapy resistance in lung squamous cell carcinoma. However, alterations in microtubules and their role in NSCLC resistance to osimertinib remain unreported. To explore the biological function of microtubules in NSCLC resistance, the microtubule morphology in osimertinib-sensitive (H1975) and -resistant (H1975OR) cells was analyzed using β-tubulin immunofluorescence staining. The results revealed significant morphological differences, including distinct perinuclear microtubule clusters uniquely observed in H1975OR cells (Fig. 1A). To determine whether this morphological change was cell line-dependent, we established another osimertinib-resistant cell line (HCC827OR) and observed a similar phenomenon (Fig. S1A). In contrast, F-actin staining of microfilaments showed no structural differences between the resistant and sensitive cells (Fig. S1B).

To investigate whether the morphological change in the resistant cells was on account of microtubule aggregation, H1975 and H1975OR cells were treated with nocodazole (a microtubule-depolymerizing agent) to assess post-washout microtubule regeneration. Results showed microtubules depolymerized in both cell lines upon nocodazole treatment, with nucleation resuming within 5 min after washout (Fig. 1A). However, after 60 min, microtubules in H1975 sensitive cells dispersed into the cytoplasm, whereas those in resistant cells remained anchored at perinuclear microtubule-organizing centers (MTOCs) (Fig. 1A). Co-staining with β-tubulin and γ-tubulin confirmed these clusters as centrosomal microtubules, indicating a marked increase in centrosomal microtubule proportion in resistant cells, particularly in H1975OR (Fig. 1B).

**Fig. 2:**
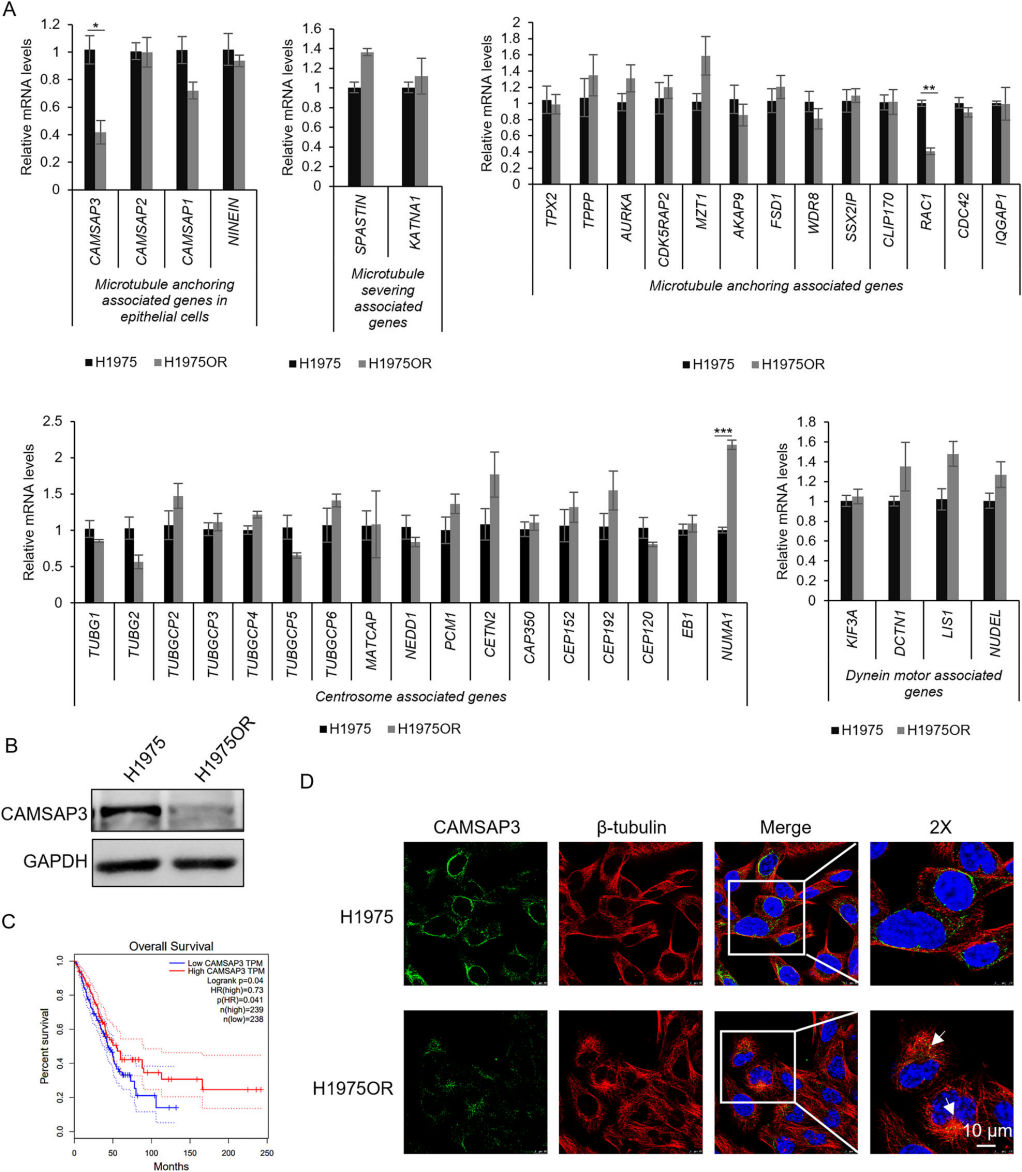
CAMSAP3 is downregulated in both H1975OR cells and patients with poor prognosis. **A** RT-qPCR analysis of expression of microtubule-related genes in H1975 and H1975OR cells (mean ± SD, n = 3), *P < 0.05, **P < 0.01, ***P < 0.001. **B** Western blot analysis to detect the CAMSAP3 protein levels in H1975 and H1975OR cells (mean ± SD, n = 3). **C** Kaplan-Meier survival analysis of lung cancer patients with high and low CAMSAP3 expression from GEPIA database. **D** Co-immunofluorescence staining for β-tubulin and CAMSAP3 in H1975/H1975OR cells, the arrowheads indicate the positions of centrosomes. DAPI stains nuclei (Scale bars, 10 μm).

To verify the effect of microtubule aggregation on the morphological differences between osimertinib-resistant and -sensitive cells, cells were treated with the depolymerizing agent nocodazole and the stabilizing agent paclitaxel. The result showed that short-term (1 h) paclitaxel or nocodazole exposure disrupted microtubules in both H1975 and H1975OR cells (Fig. S1C). Prolonged treatment (48 h) resulted in a more pronounced reduction in cell viability and survival in H1975OR and HCC827OR cells compared to their sensitive counterparts (Fig. 1C, D), suggesting heightened sensitivity of resistant cells to microtubule-targeting agents. To investigate the underlying mechanisms of the morphological difference in osimertinib-resistant cells, we analyzed transcriptional levels of microtubule associated genes including *CAMSAP1/2/3*, *NINEIN*, *TPX2*, etc., microtubule dynamics genes including *SPASTIN*, *KIF3A*, *DCTN1*, etc., and centrosome function genes including *TUBG1*, *NEDD1*, *NUMA1*, etc. (Fig. 2A). Results showed that *CAMSAP3* mRNA levels decreased by nearly 60% in osimertinib-resistant cells compared to sensitive cells, indicating that CAMSAP3 may play an important role in centrosomal/non-centrosomal microtubule regulation.

### CAMSAP3 contributes to osimertinib resistance in NSCLC

To further validate the role of CAMSAP3 in NSCLC cell resistance to osimertinib, protein expression of CAMSAP3 in H1975OR cells was further assessed by western blot and immunofluorescence staining. Consistent with RT-qPCR results, CAMSAP3 protein levels were significantly reduced in H1975OR cells (Fig. 2B). Moreover, Kaplan-Meier survival analysis revealed shorter overall survival in lung adenocarcinoma patients with low CAMSAP3 levels, implicating the prognostic relevance of CAMSAP3 expression in lung cancer progression (Fig. 2C). Next, the localization of CAMSAP3 in NSCLC cells was assessed. As shown in Fig. 2D, co-immunofluorescence staining of CAMSAP3 and β-tubulin demonstrated uniform perinuclear CAMSAP3 distribution with predominant non-centrosomal microtubule organization in sensitive H1975 cells. In contrast, osimertinib-resistant cells displayed reduced CAMSAP3 expression and retained centrosomal microtubule clustering, a pattern replicated in HCC827OR cells (Fig. S2A–C). These findings suggested that the centrosomal microtubule aggregation regulator CAMSAP3 is dramatically decreased in osimertinib-resistant NSCLC, which may affect lung cancer progression.

### CAMSAP3 modulates osimertinib sensitivity and microtubule morphology

**Fig. 3:**
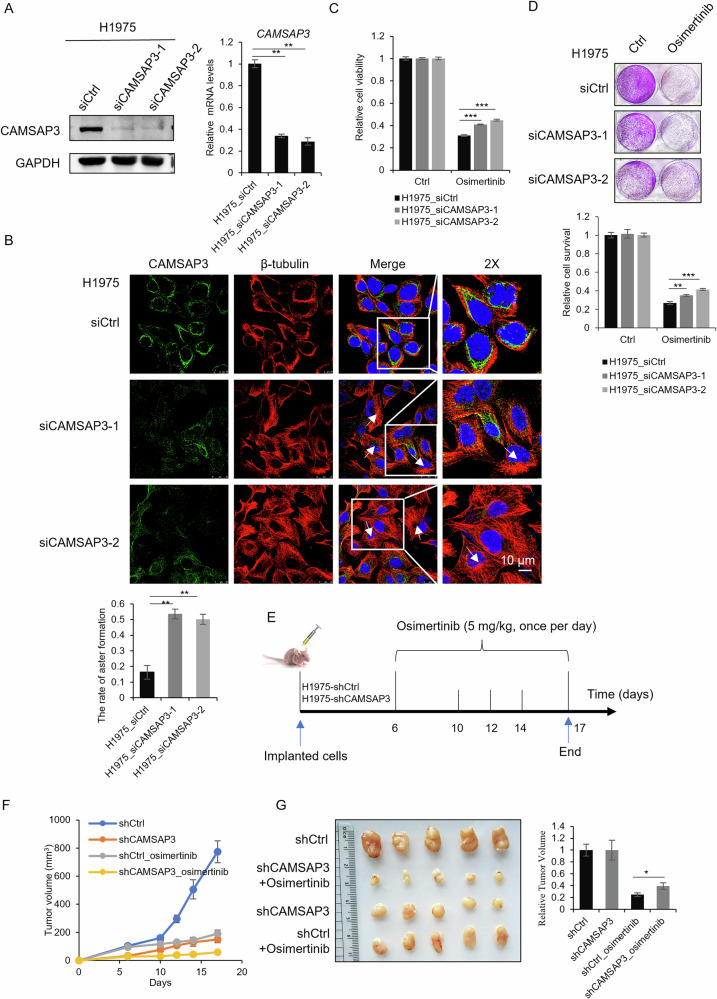
CAMSAP3 knockdown recapitulates the altered microtubule formation and drug sensitivity seen in osimertinib-resistant cells. **A** CAMSAP3 knockdown efficiency in H1975 cells with two independent siRNAs (mean ± SD, n = 3), **P < 0.01. **B** Co-immunofluorescence staining of β-tubulin and CAMSAP3 in H1975 cells transfected with the indicated siRNAs. The arrowheads indicate the position of centrosomes. DAPI stains nuclei (Scale bars, 10 μm). The number of cells with centrosomal microtubules in (**B**) was quantified, in which 100 cells were analyzed per experiment, **P < 0.01. **C** The cell viabilities of CAMSAP3-silenced H1975 cells were detected by MTT assay after being treated with osimertinib for 48 h (mean ± SD, n = 6), ***P < 0.001. **D** Crystal violet staining assay was performed for CAMSAP3-silenced H1975 cells after being treated with osimertinib for 48 h (mean ± SD, n = 3), **P < 0.01, ***P < 0.001. **E** Diagram of a mouse xenograft treated with osimertinib. **F** Tumor volume was measured on days 6, 10, 12, 14, and 17 after implantation. **G** The tumors were dissected and photographed at the end of the experiment. The tumor volume inhibition (based on the final tumor volume) in (**G**) was quantified (mean ± SD, n = 5), *P < 0.05.

After establishing the role of CAMSAP3 in microtubule aggregation, the biological function of CAMSAP3 in NSCLC drug resistance was then assessed. CAMSAP3 was knocked down by two independent siRNAs in H1975 and HCC827 cells. As shown in Fig. 3A and Fig. S3A, CAMSAP3 knockdown efficiency was verified at both protein and mRNA levels. For microtubule morphology, co-immunofluorescence staining of CAMSAP3 and β-tubulin revealed centrosomal microtubule clustering in CAMSAP3-depleted H1975 (Fig. 3B) and HCC827 cells (Fig. S3B), consistent with our hypothesis. Then, MTT and crystal violet assays demonstrated increased cell viability and staining intensity in CAMSAP3-knockdown H1975 (Fig. 3C, D) and HCC827 cells (Fig. S3C, S3D) after osimertinib treatment, indicating that CAMSAP3 silencing confers resistance to osimertinib. In order to validate these findings in vivo, H1975 cells stably expressing control shRNA (shCtrl) or shCAMSAP3 underwent xenograft assays (Fig. S3E). β-tubulin staining confirmed enhanced microtubule clustering in CAMSAP3-silenced H1975 cells (Fig. S3F). Nude mice were treated with 5 mg/kg osimertinib when the largest subcutaneous tumor volumes reached ~100 mm³. Tumor volumes were monitored from post-implantation until tissue harvest on designated days (Fig. 3E). As shown in Fig. 3F, G, osimertinib reduced tumor volumes in both groups, while the final tumor volume inhibition revealed that CAMSAP3 knockdown significantly increased tumor resistance to osimertinib compared with the control group (Fig. 3G), confirming the critical role of CAMSAP3 in modulating osimertinib resistance.

**Fig. 4:**
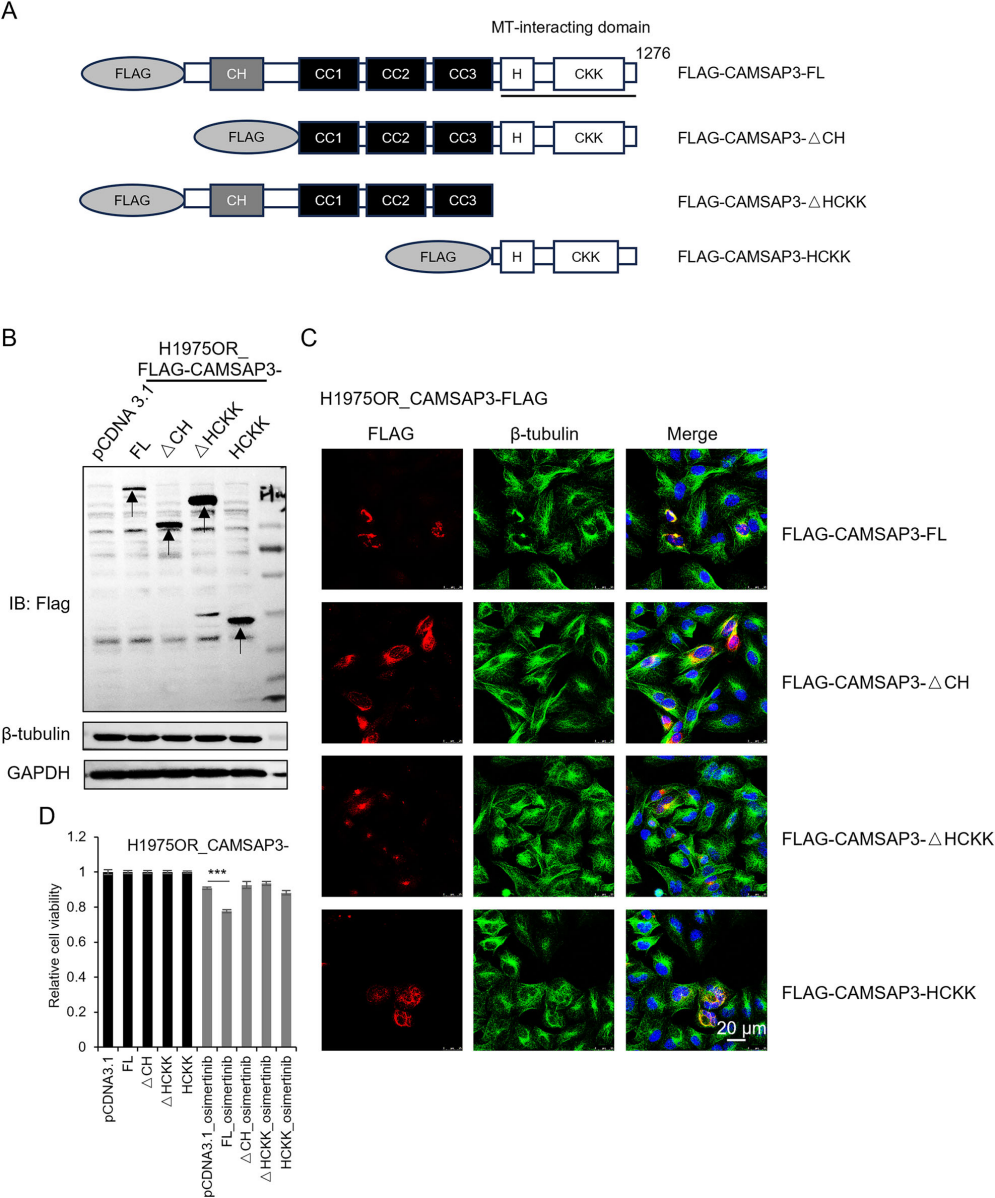
Overexpression of CAMSAP3 in osimertinib-resistant cells rescues the altered microtubule formation and enhances the drug sensitivity. **A** Structures of the full-length CAMSAP3 (CAMSAP3-FL) and its three truncated forms (CAMSAP3-ΔCH; CAMSAP3-ΔHCKK; CAMSAP3-HCKK), tagged with FLAG. The FLAG region is used as the antigen for generating anti-CAMSAP3 antibodies. **B** Western blot analysis of CAMSAP3 (CAMSAP3-FL) and its three truncated forms expressed in H1975OR cells (mean ± SD, n = 3). The arrowheads indicate expected protein sizes positions. **C** Co-immunofluorescence staining of FLAG and β-tubulin in H1975OR cells transfected with FLAG-tagged CAMSAP3 and its three truncated form plasmids. DAPI stains nuclei (Scale bars, 20 μm). **D** The cell viability of CAMSAP3 and its three truncated forms overexpressed in H1975OR cells after being treated with 1 μM osimertinib for 48 h was measured by MTT assays (mean ± SD, n = 6), ***P < 0.001.

CAMSAP3 comprises various functional domains. To determine which domain mediates microtubule remodeling and drug resistance regulation, different truncated variants of CAMSAP3 were overexpressed in osimertinib-resistant cells, including FLAG-tagged: Full-Length CAMSAP3 (CAMSAP3-FL), Truncations Lacking the CH Domain (CAMSAP3-ΔCH), Truncations Lacking the HCKK Domain (CAMSAP3-ΔHCKK), and an HCKK-Only Construct (CAMSAP3-HCKK) (Fig. 4A). Microtubule morphology analysis revealed that only CAMSAP3-FL partially restored non-centrosomal microtubule distribution in H1975OR cells (Fig. 4B, C), although cytoplasmic parallel microtubule organization (as in sensitive cells; Fig. 1A) was not fully restored. CAMSAP3-ΔHCKK mislocalized to microtubule-organizing centers (MTOCs), highlighting the HCKK domain’s necessity for proper CAMSAP3 localization (Fig. 4C). The impact of these truncations on osimertinib sensitivity was then assessed. MTT assays showed that CAMSAP3-FL overexpression restored drug sensitivity in resistant cells compared to truncated variants (Fig. 4D). Notably, transient CAMSAP3-FL transfection induced significantly higher cell death ratio, so a stable overexpression of CAMSAP3-FL cell line could not be established. These findings indicate that CAMSAP3 overexpression disrupts microtubule networks and restores resistant cells’ sensitivity to osimertinib.

### CAMSAP3 disrupts lysosomal and EGFR localization

**Fig. 5:**
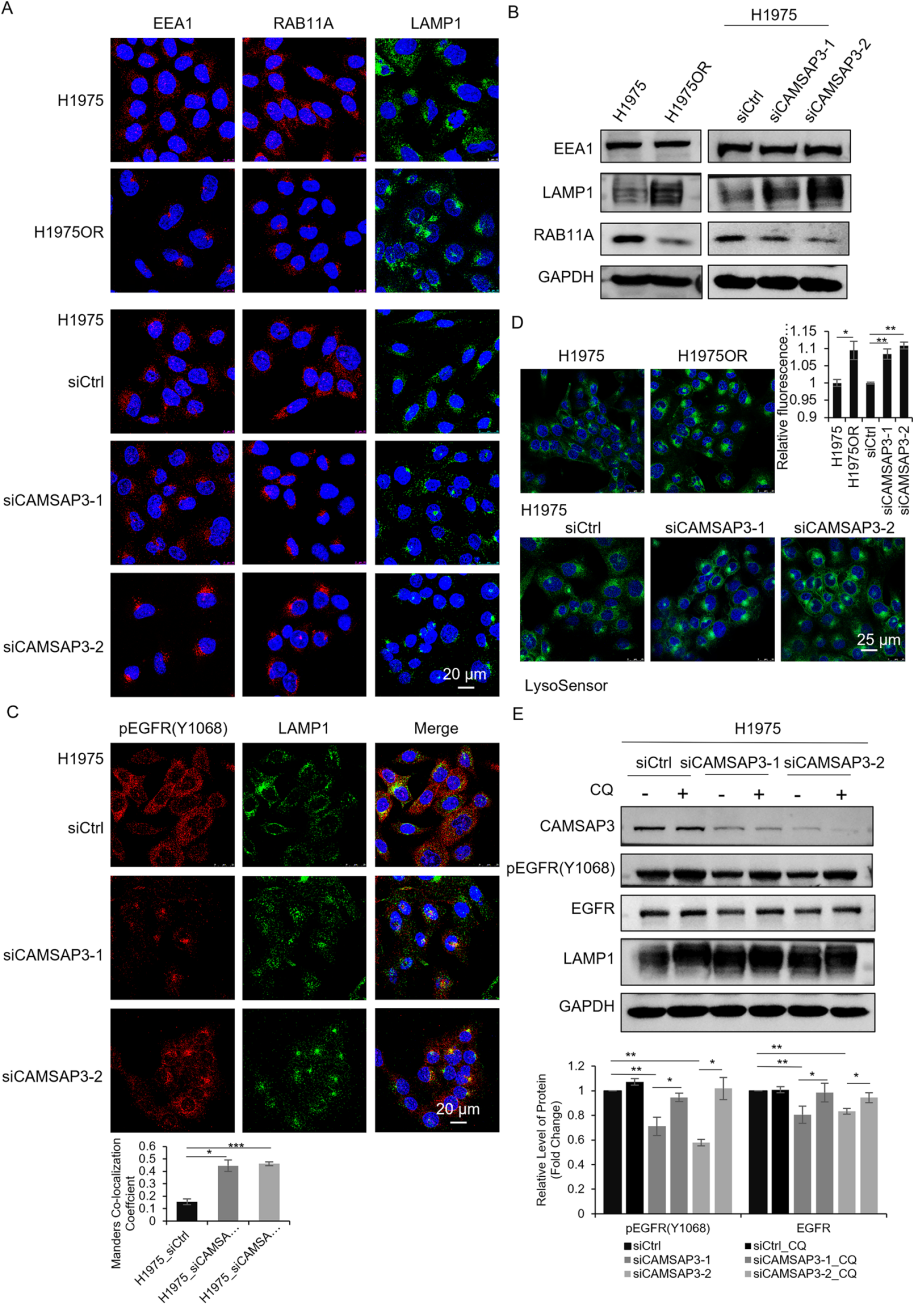
CAMSAP3 knockdown recapitulates the endosome-lysosome dislocation seen in osimertinib-resistant cells. **A** Immunofluorescence staining for EEA1, RAB11A and LAMP1 in H1975/H1975OR and H1975 cells transfected with two independent siRNAs. DAPI stains nuclei (Scale bars, 20 μm). **B** Western blot analysis of EEA1, RAB11A and LAMP1 protein levels in H1975/H1975OR and H1975 cells transfected with two independent siRNAs. (mean ± SD, n = 3). **C** Co-immunofluorescence staining for LAMP1 and pEGFR(Y1068) in H1975 cells transfected with the indicated siRNAs. DAPI stains nuclei (Scale bars, 20 μm). The colocalization of LAMP1 and pEGFR(Y1068) was quantified using Manders’ colocalization coefficients, *P < 0.05, ***P < 0.001. **D** Lysosomal pH was detected by LysoSensor Green DND-189 in H1975/H1975OR and H1975 cells transfected with two independent siRNAs. Hoechst 33342 was used to stain nuclei. (Scale bars, 25 μm). Relative fluorescence intensity in (**D**) was quantified, in which 50 cells were analyzed per experiment, *P < 0.05, **P < 0.01. **E** Western blot analysis of EGFR and pEGFR(Y1068) levels in H1975 cells transfected with the indicated siRNAs with or without 25 μM CQ treatment for 48 h (mean ± SD, n = 3). Band intensities were normalized to GAPDH, *P < 0.05, **P < 0.01.

EGFR degradation and downregulation represent critical factors contributing to osimertinib resistance, with the lysosomal pathway playing a key role in this process. However, the relationship between microtubule-driven lysosomal alterations and osimertinib resistance remains unexplored. It is well established that the early endosomes (marked by EEA1), lysosomes (LAMP1), and recycling endosomes (RAB11A) are the primary regulators of lysosomal trafficking. Therefore, we examined their distribution in H1975 and H1975OR cells. Immunofluorescence revealed cytoplasmic distribution of EEA1, LAMP1, and RAB11A in sensitive H1975 cells, whereas all three organelles were clustered at perinuclear microtubule-organizing centers (MTOCs) in H1975OR and CAMSAP3-knockdown cells (Fig. 5A). Western blotting showed reduced RAB11A levels and elevated LAMP1 levels in H1975OR and CAMSAP3-knockdown cells (Fig. 5B), suggesting that CAMSAP3 deficiency alters endo-lysosomal trafficking and lysosomal protein expression.

**Fig. 6:**
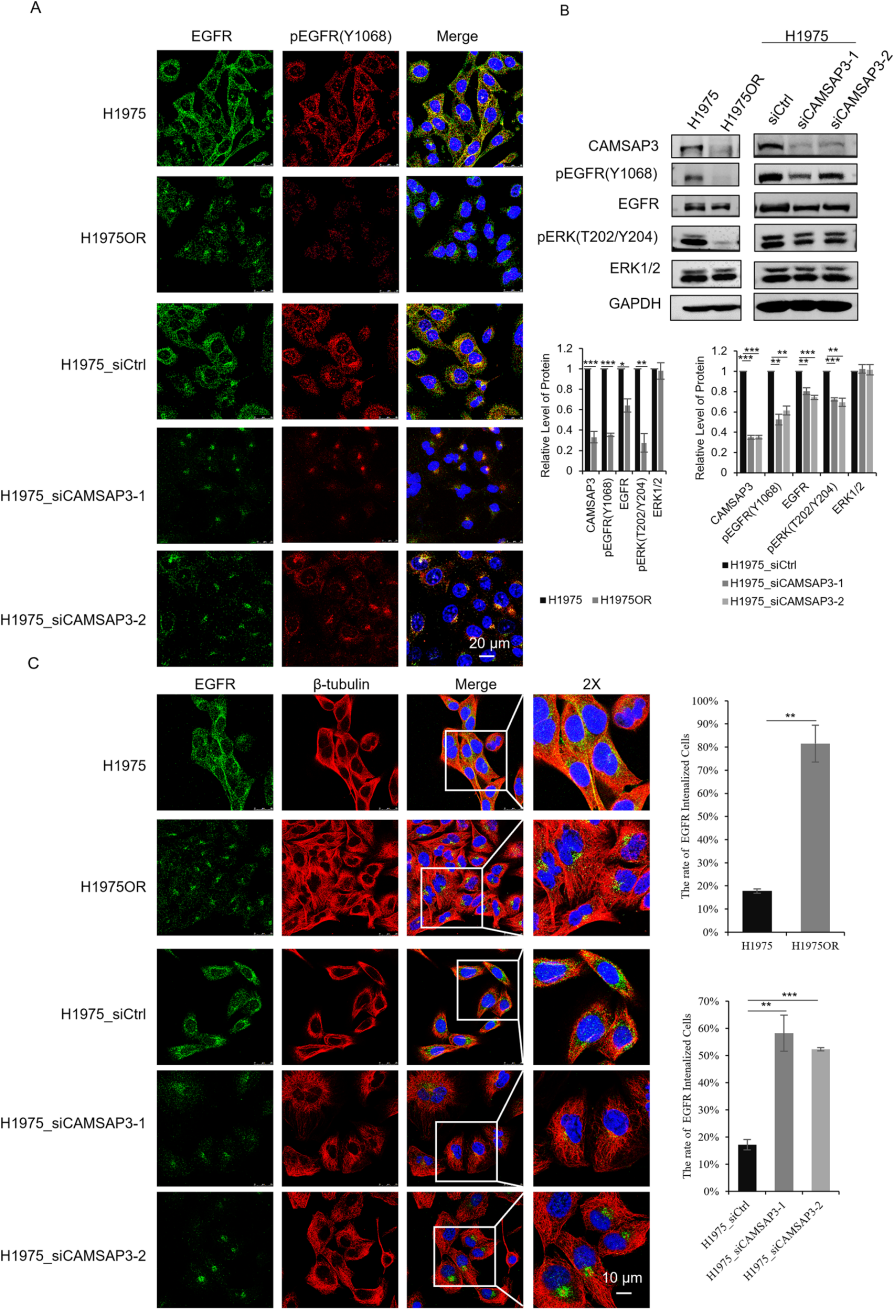
CAMSAP3 knockdown recapitulates the EGFR dislocation seen in osimertinib-resistant cells. **A** Co-immunofluorescence staining of EGFR and pEGFR(Y1068) in H1975/H1975OR and H1975 cells transfected with two independent siRNAs, DAPI stains nuclei (Scale bars, 20 μm). **B** Western blot analysis of EGFR and pEGFR(Y1068), ERK1/2 and pERK1/2(T202/Y204) in H1975/H1975OR and H1975 cells transfected with siRNAs (mean ± SD, n = 3). Band intensities were normalized to GAPDH, *P < 0.05, **P < 0.01, ***P < 0.001. **C** Co-immunofluorescence staining of EGFR and β-tubulin in H1975/H1975OR and H1975 cells transfected with two independent siRNAs. DAPI stains nuclei (Scale bars, 10 μm). The number of cells with internalization of EGFR into the MTOC in (**C**) was quantified, in which 50 cells were analyzed per experiment, **P < 0.01, ***P < 0.001.

Mutant EGFRs (exon 19 deletions, T790M/L858R) are typically resistant to degradation due to ubiquitination/lysosomal defects. Surprisingly, EGFR and pEGFR (Y1068) levels were greatly reduced in both osimertinib-resistant and CAMSAP3-knockdown cells (Fig. 6B, S5B). Meanwhile, co-immunofluorescence staining revealed enhanced colocalization of pEGFR(Y1068) and LAMP1 upon CAMSAP3 knockdown in H1975 (Fig. 5C) and HCC827 cells (Fig. S4A), demonstrating CAMSAP3-associated lysosomal localization of pEGFR. Lysosomal acidification is an important factor of lysosomal activity during protein degradation. LysoSensor Green DND-189 staining (a pH-sensitive indicator) showed increased lysosomal acidity in H1975OR and CAMSAP3-knockdown cells (Fig. 5D), verifying the role of CAMSAP3 in lysosome degradation activity. Chloroquine (CQ) was used to inhibit lysosomal activity. Western blotting revealed that CQ prevented the degradation of EGFR and pEGFR(Y1068) induced by CAMSAP3 knockdown in H1975 and HCC827 cells (Fig. 5E, S4B), confirming that loss of CAMSAP3 promoted lysosomal degradation of mutant EGFR.

To assess the potential of CQ to inhibit osimertinib-resistant cell growth, osimertinib-resistant and -sensitive cells were treated with CQ either alone or in combination with osimertinib for 48 h. These experiments revealed that the combination of CQ and osimertinib enhanced efficacy in both resistant and sensitive cells. Furthermore, CQ was more toxic to resistant cells than to sensitive cells (Fig. S4C, S4D). Xenograft models confirmed that the CQ-osimertinib combination treatment significantly enhanced tumor growth inhibition in osimertinib-resistant tumors (Fig. S4E, S4F). These data demonstrate that CQ enhances the efficacy of osimertinib and suppresses the growth of osimertinib-resistant cells both in vitro and in vivo.

Given that EGFR trafficking is microtubule-regulated, the localization of EGFR/pEGFR(Y1068) in osimertinib-resistant and sensitive cells was examined next. As shown in Fig. 6A and Fig. S5A, EGFR/pEGFR predominantly localized to the plasma membrane in sensitive cells, whereas in resistant cells, it accumulated at perinuclear MTOCs with reduced expression levels. Continuous osimertinib treatment resulted in undetectable pEGFR in resistant cells. CAMSAP3 knockdown similarly shifted EGFR/pEGFR to perinuclear MTOCs in both H1975 and HCC827 cells (Fig. 6A, S5A). Western blotting confirmed reduced expression of EGFR, pEGFR, and pERK1/2(T202/Y204) in osimertinib-resistant and CAMSAP3 knockdown cells compared to sensitive controls (Fig. 6B, S5B), affirming the role of CAMSAP3 in EGFR degradation. However, EGFR transcriptional levels remained unchanged in osimertinib-resistant and CAMSAP3 knockdown cells (Fig. S5C and S5D), suggesting EGFR loss is exclusively regulated through post-translational degradation.

## Discussion

Osimertinib has become the first-line treatment choice for EGFR-mutant NSCLC, with greater efficacy and improved overall survival compared to the previous generation of EGFR TKIs [8, 26]. The interaction of the microtubule system with tyrosine kinase (TK) signaling pathways plays a key role in tumor drug resistance [27]. Tubulin-binding agents (e.g., paclitaxel, docetaxel) inhibit microtubule dynamics by targeting β-tubulin in α/β-tubulin heterodimers, inducing mitotic arrest and apoptosis. These agents, particularly paclitaxel, form the backbone of first-line NSCLC therapy, administered either alone or combined with platinum-based chemotherapy (cisplatin/carboplatin) [28, 29]. Preclinical evidence demonstrates that chronic EGFR inhibition triggers non-genetic resistance through TPX2-mediated Aurora kinase A (AURKA) activation; thus, combining an AURKA inhibitor with third-generation EGFR-TKI osimertinib can overcome acquired resistance in NSCLC [30]. TPX2 and AURKA are involved in microtubule nucleation/mitotic spindle assembly [31, 32], and our study revealed distinct microtubule localization patterns between drug-sensitive and resistant cells. In sensitive cells, microtubules were released into the cytoplasm following nucleation, whereas in osimertinib-resistant cells, microtubules remained anchored in the perinuclear region post-nucleation. These findings suggest that altered microtubule dynamics may contribute to TKI resistance. Studying microtubule dynamics in drug-resistant cells is an important approach to understanding and overcoming drug resistance.

The EGFR signaling pathway is a central driver of tumor growth, but inhibitors like osimertinib often face resistance [33]. NSCLC EGFR TKI resistance is classically linked to secondary EGFR mutations (e.g., T790M/C797S). Over 40% of EGFR TKI resistance cases now originate from tumor cells abandoning EGFR dependency through: (1) Bypass activation, including MET amplification-mediated ERBB3-PI3K/AKT signaling, HER2/HER3 heterodimer-driven MAPK activation and AXL-RTK-mediated EMT [34–36], or (2) Downstream mutations (e.g., KRAS G12C/BRAF V600E) that bypass EGFR regulation [37], or (3) Phenotypic plasticity through tumor stem cell maintenance via WNT/Notch pathways or metabolic reprogramming (e.g., glutamine dependency) [38, 39]. These mechanisms illustrate that EGFR TKI resistance often emerges when cells survive in an EGFR-independent manner. Previous studies have demonstrated that osimertinib promotes EGFR degradation [40]. Consistent with these findings, we observed EGFR degradation in osimertinib-resistant cells, suggesting that EGFR may become dispensable for resistant cell survival. However, the precise molecular mechanisms regulating this degradation process are not fully elucidated. Therefore, further investigation is warranted to elucidate the mechanisms underlying EGFR degradation in drug-resistant cells.

**Fig. 7:**
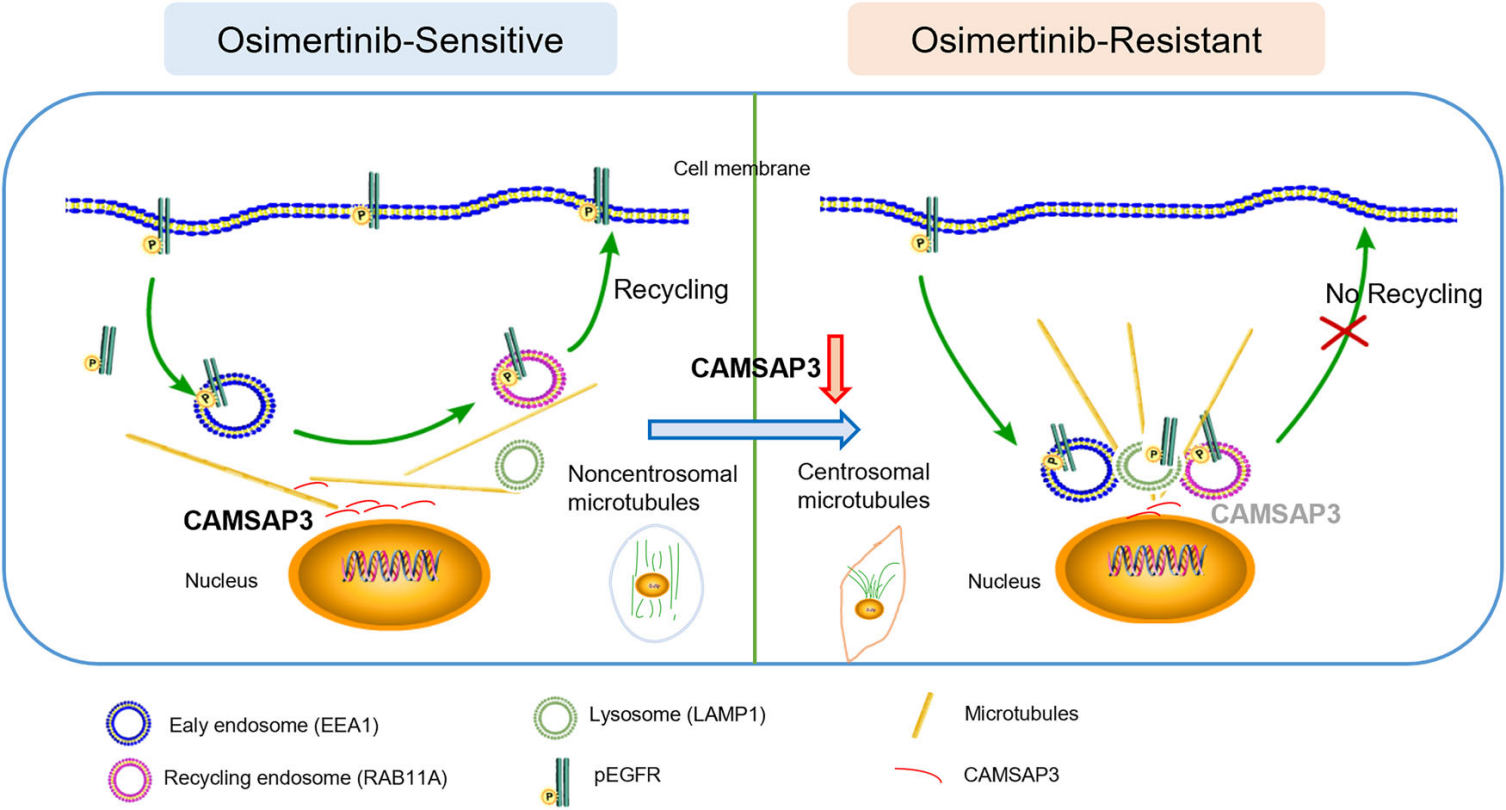
Schematic representation of CAMSAP3-regulated different distribution of microtubules as well as endosome-lysosomes in osimertinib-sensitive and -resistant NSCLC cells. CAMSAP3 knockdown in osimertinib-sensitive cells resulted in a significant increase in the proportion of non-centrosomal microtubules, which in turn altered the internalization/degradation pathway of EGFR/pEGFR, preventing them from recycling to the cytoplasmic membrane. This is similar to what happens in osimertinib-resistant cells.

EGFR, a transmembrane receptor tyrosine kinase, undergoes EEA1-mediated endocytosis followed by lysosomal degradation or plasma membrane recycling [41, 42]. Mutant EGFR evades CBL-mediated ubiquitination and lysosomal degradation, leading to persistent activation of EGFR signaling [13]. Single-cell sequencing further reveals that EGFR-independent clones in the tumor microenvironment propagate resistance via exosomal miRNAs, underscoring the need to target the tumor ecosystem in TKI-resistant cells [43, 44]. In this study, we observed perinuclear accumulation of EGFR at microtubule organizing centers (MTOCs) in osimertinib-resistant NSCLC cells. Mechanistic investigations revealed that CAMSAP3, a microtubule-remodeling protein, promotes EGFR depletion through lysosomal degradation. Specifically, CAMSAP3 knockdown induced aberrant non-centrosomal microtubule assembly, which disrupted normal EGFR trafficking in sensitive cells. This disruption diverted EGFR/pEGFR from the plasma membrane recycling pathway to lysosomal degradation (Fig. 7). These findings demonstrate that loss of CAMSAP3 induces NSCLC cell growth in an EGFR-independent manner, leading to the acquired resistance to osimertinib.

Collectively, our functional evidence establishes CAMSAP3 deficiency as an active driver of osimertinib resistance. Genetic modulation of CAMSAP3 expression (through silencing or overexpression) directly altered microtubule architecture and, crucially, reduced or restored sensitivity to osimertinib in sensitive or resistant cells. Critically, published evidence links CAMSAP3 loss to cytoskeleton-dependent mechanisms like EMT [45] and angiogenesis [25], reinforcing its causal role. We demonstrate that loss of CAMSAP3 confers resistance to osimertinib through EGFR-independent pathways in NSCLC, though the precise molecular mechanisms underlying CAMSAP3-mediated acquired resistance require further exploration using omics approaches.

The findings of this study demonstrate that osimertinib-resistant cells exhibit increased sensitivity to paclitaxel and chloroquine (CQ), indicating that a combination therapy consisting of osimertinib, microtubule-stabilizing agents (e.g., paclitaxel), and autophagy-lysosome inhibitors (e.g., CQ) constitutes a rational therapeutic strategy for clinical trials targeting CAMSAP3-low NSCLC. In addition to our prognostic data concerning lung cancer, emerging evidence suggests a link between CAMSAP3 loss and therapy resistance. For instance, low CAMSAP3 expression has been associated with more aggressive behavior and poor prognosis in early-stage endometrial cancer [46], reinforcing its broad role as a resistance driver. This multi-cancer relevance strengthens its potential as a biomarker.

## Supplementary information

Supplementary materials

Original western blots

## Data Availability

All data needed to evaluate the conclusions in the paper are present in the paper and/or the Supplementary Materials.
