# Supplementary material for: Cytoskeletal remodeling via CAMSAP3 downregulation drives resistance to osimertinib in NSCLC cells
Source: Cell Death Dis. 2025 Dec 11;17(1):90. doi: 10.1038/s41419-025-08299-0 (PMC12830379; doi:10.1038/s41419-025-08299-0)
Supplement: Supplementary file 1 — Supplementary materials [file 41419_2025_8299_MOESM1_ESM.pdf]

The PDF file includes:

Fig. S1 to S5

Supplementary Tables S1 to S4

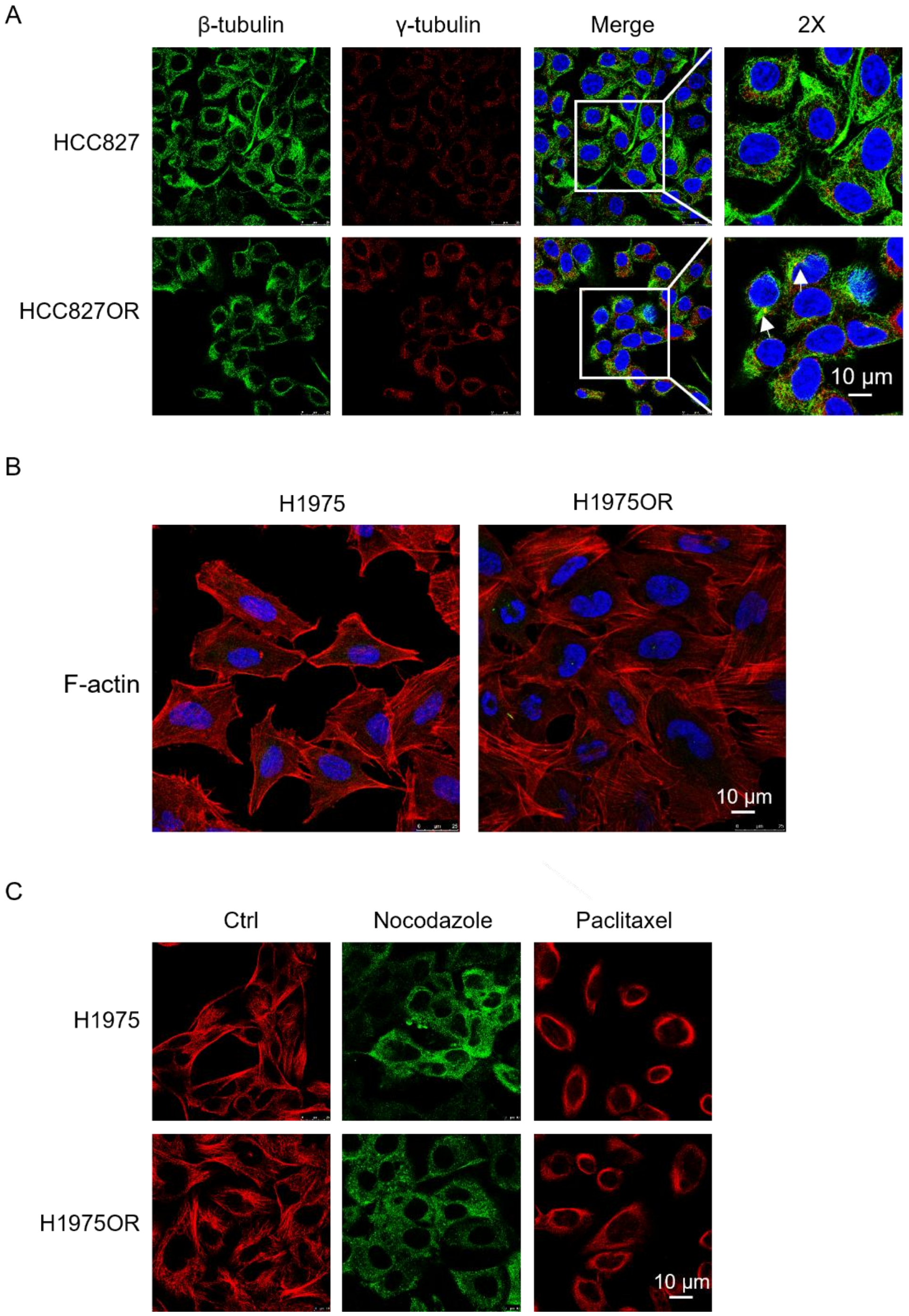

Fig. S1 Representative immunofluorescence staining in H1975/H1975OR and HCC827/HCC827OR cells.

(A) Immunofluorescence staining for β-tubulin and γ-tubulin in HCC827/HCC827OR cells.

Centrosomes were stained with anti-γ-tubulin antibodies. The arrowheads indicate centrosomal positions. DAPI stains nuclei (Scale bars, 10 μm). (B) Immunofluorescence staining for F-actin in H1975/H1975OR cells. DAPI stains nuclei (Scale bars, 10 μm). (C) Immunofluorescence staining for β-tubulin in H1975 and H1975OR cells treated with nocodazole (1 μg/mL) and paclitaxel (1 μg/mL) for 1 h (Scale bars, 10 μm).

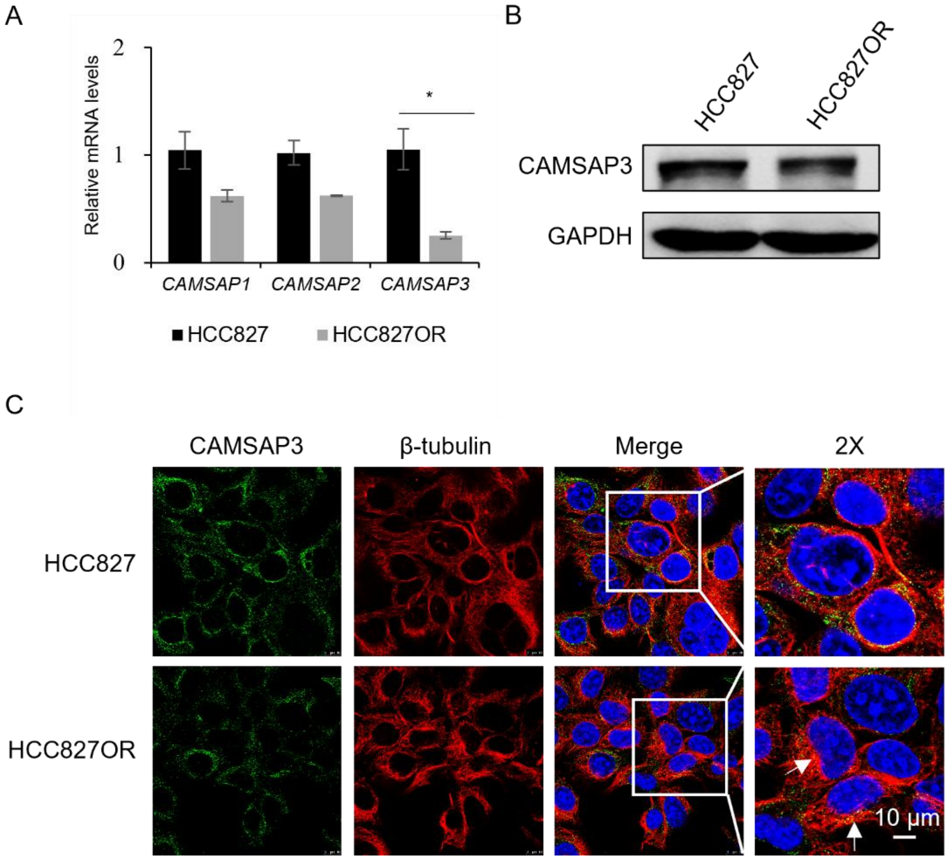

Fig. S2 Identification of CAMSAP3 expression by RT-qPCR, western blot and immunofluorescence staining in HCC827/HCC827OR cells.

(A) RT-qPCR analysis of *CAMSAP1/2/3* mRNA levels in HCC827 and HCC827OR cells (mean ± SD, n = 3), *$P < 0.05$. (B) Western blot analysis of CAMSAP3 protein levels in HCC827 and HCC827OR cells (mean ± SD, n = 3). (C) Co-immunofluorescence staining for β-tubulin and CAMSAP3 in HCC827/HCC827OR cells. The arrowheads indicate centrosomal positions. DAPI stains nuclei (Scale bars, 10 μm).

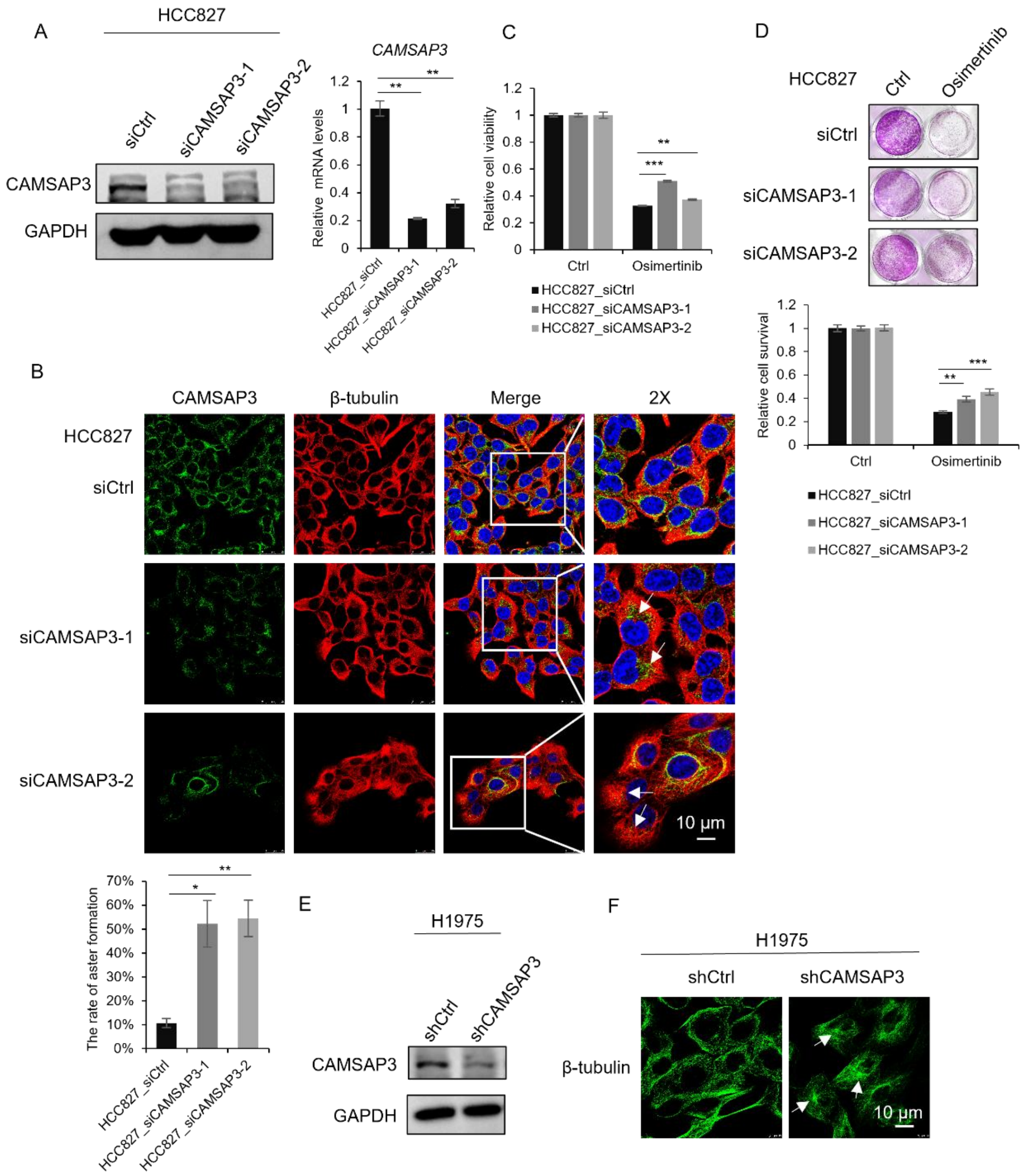

Fig. S3 Identification of CAMSAP3 knockdown efficiency in HCC827 cells.

(A) CAMSAP3 knockdown efficiency in HCC827 cells with the indicated siRNAs (mean ± SD, n = 3), **P < 0.01. (B) Co-immunofluorescence staining for β-tubulin and CAMSAP3 in HCC827 cells transfected with the indicated siRNAs. The arrowheads indicate centrosomal positions. DAPI stains nuclei (Scale bars, 10 μm). The number of cells with centrosomal microtubules in Fig. S3B was quantified, in which 100 cells were analyzed per experiment, *P < 0.05, **P < 0.01. (C) After silencing CAMSAP3 with the specific siRNA for 24 h in HCC827 cells, followed by treatment with 0.1 μM osimertinib for 48 h, cell viability was detected by MTT assay (mean ± SD, n = 6), **P <

0.01, ****P* < 0.001. (D) After silencing CAMSAP3 with two independent siRNAs for 24 h in HCC827 cells, followed by treatment with 0.1 μM osimertinib for 48 h, the cell survival was detected by crystal violet staining (mean ± SD, n = 3), ***P* < 0.01, ****P* < 0.001. (E) Stable CAMSAP3 knockdown efficiency with the indicated shRNA in H1975 cells. (F) Immunostaining for β-tubulin in H1975_shCtrl and H1975_shCAMSAP3 cells. The arrowheads indicate centrosomal positions (Scale bars, 10 μm).

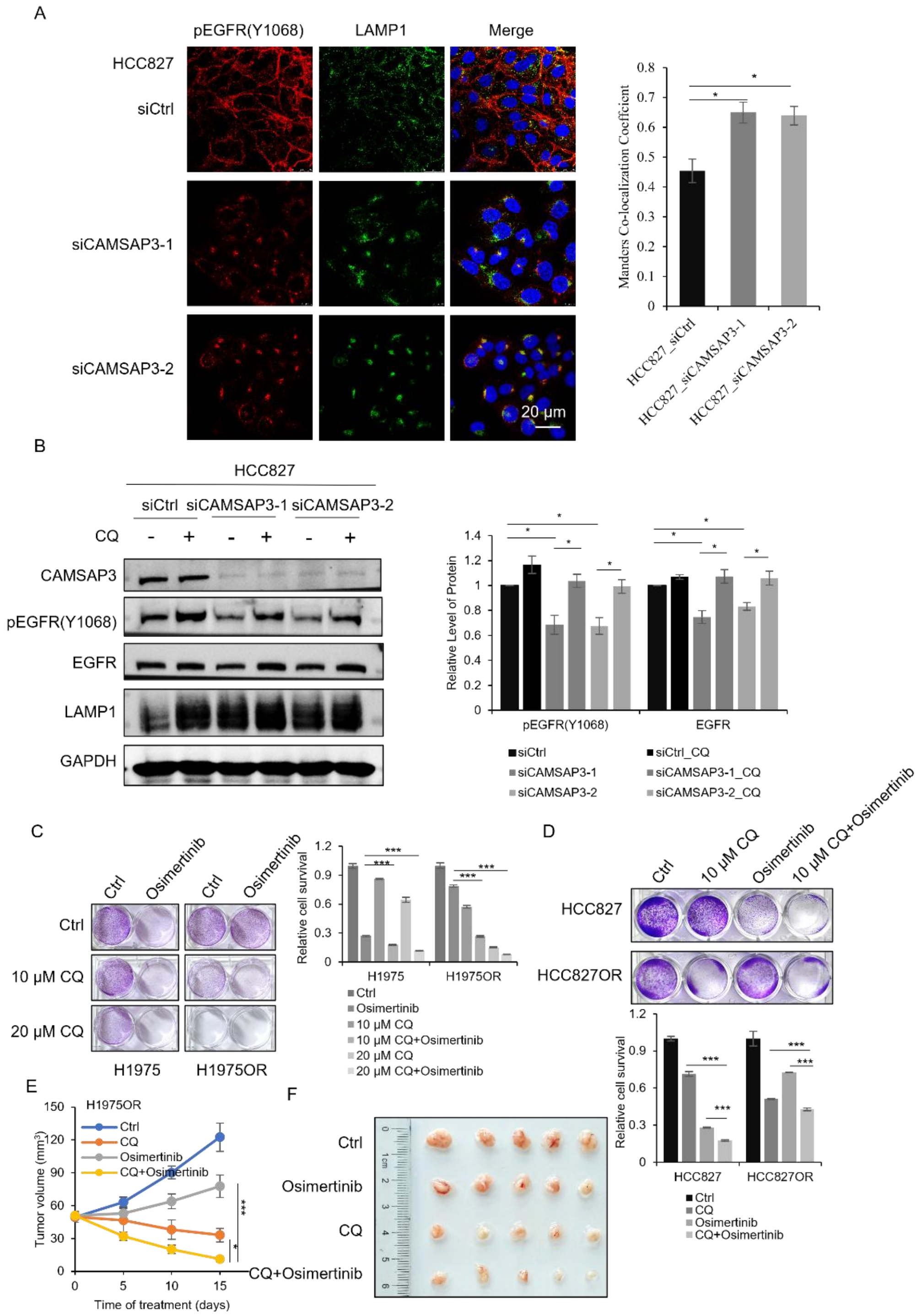

Fig. S4 CQ-osimertinib combination suppresses the growth of osimertinib-resistant cells.

(A) Co-immunofluorescence staining for LAMP1 and pEGFR(Y1068) in HCC827 cells transfected with the indicated siRNAs. DAPI stains nuclei (Scale bars, 20 μm). Colocalization of LAMP1 and pEGFR(Y1068) was quantified as Manders colocalization coefficients, $*P < 0.05$. (B) Western blot analysis of EGFR and pEGFR(Y1068) levels in HCC827 cells transfected with the indicated siRNAs with or without 25 μM CQ treatment for 48 h (mean ± SD, n = 3). Band intensities were measured and normalized to GAPDH bands, $*P < 0.05$. (C) Crystal violet staining assay was performed on H1975 and H1975OR cells after being treated with 10 μM and 20 μM CQ and/or 1 μM osimertinib for 48 h (mean ± SD, n = 3), $***P < 0.001$. (D) Crystal violet staining assay was performed on HCC827 and HCC827OR cells after being treated with 10 μM CQ and/or 1 μM osimertinib for 48 h (mean ± SD, n = 3), $***P < 0.001$. (E) Tumor volume was measured on the indicated days (mean ± SD, n = 5), $*P < 0.05$, $***P < 0.001$. (F) Tumors were dissected and photographed at the end of the experiment.

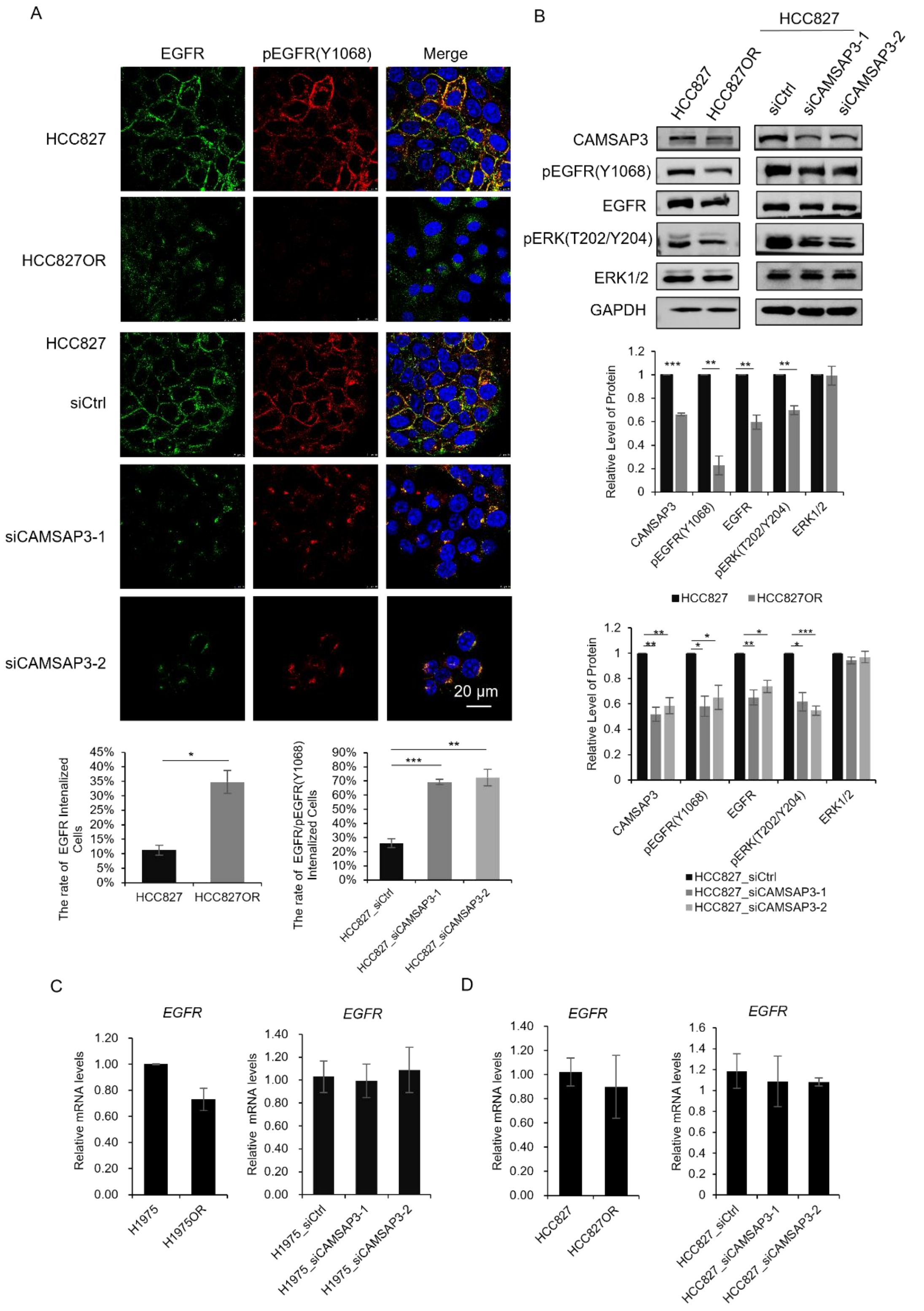

Fig. S5 Identification of the mRNA level and protein level of EGFR in HCC827/HCC827OR cells.

(A) Double immunostaining for EGFR and pEGFR(Y1068) in HCC827/HCC827OR and HCC827

cells transfected with the indicated siRNAs. DAPI stains nuclei (Scale bars, 20 μm). The number of cells with internalization of EGFR into the MTOC in Fig. S5A was quantified, in which 50 cells were analyzed per experiment, $^{*}P < 0.05$, $^{**}P < 0.01$, $^{***}P < 0.001$. (B) Western blot analysis of EGFR and pEGFR(Y1068), ERK1/2 and pERK1/2(T202/Y204) levels in HCC827/HCC827OR and HCC827 cells transfected with the indicated siRNAs (mean ± SD, n = 3). Band intensities were measured and normalized to GAPDH bands, $^{*}P < 0.05$, $^{**}P < 0.01$, $^{***}P < 0.001$. (C) RT-qPCR analysis of *EGFR* mRNA levels in H1975/H1975OR and H1975 cells transfected with the indicated siRNAs (mean ± SD, n = 3). (D) RT-qPCR analysis of *EGFR* mRNA levels in HCC827/HCC827OR and HCC827 cells transfected with the indicated siRNAs (mean ± SD, n = 3).

Supplementary Tables:

Supplementary Table S1. The primers used for real-time PCR.

Gene	Sequences (5'-3')	
CAMSAP3	Forward	CACCATCCACTGCTATTGTCCC
CAMSAP3	Reverse	AATCCTGCACGAGCTGGAGGTT
CAMSAP2	Forward	GGAGGTCAAAAGGCTCGTTATCG
CAMSAP2	Reverse	GGCAGCTAATGCACAGCCATCT
CAMSAP1	Forward	ACCCGAGTTGATGGTCAGCCTC
CAMSAP1	Reverse	CACAACTCGCAGCGTGATGTAG
NINEIN	Forward	AGAGTGCTCAGGCTTCCGTTGA
NINEIN	Reverse	ATGACCAGCTCTGCCTCAATGC
SPAST	Forward	CTCTTTGGTCCACCTGGGAATG
SPAST	Reverse	GCCACAGCAAAAAGAGCCCTCA
KATNA1	Forward	GTGCGGACATTACCAACGTGTG
KATNA1	Reverse	CCTCCATAGTTGTAGGCATGTGC
TPX2	Forward	TTCAAGGCTCGTCCAAACACCG
TPX2	Reverse	GCTCTCTTCTCAGTAGCCAGCT
TPPP	Forward	TGACCGTCACTGACGTGGACAT
TPPP	Reverse	CGCTGCTCTTGTCTTTGAATCGC
AURKA	Forward	GCAACCAGTGTACCTCATCCTG
AURKA	Reverse	AAGTCTTCCAAAGCCCACTGCC
CDK5RAP2	Forward	ACCGATCAACACTGCACTCAGC
CDK5RAP2	Reverse	GGATTGGCAAGCGGGACTTCTT
MZT1	Forward	GTGTACGGCTTTGTGAACAAGGA
MZT1	Reverse	GAATTTCTCCAGAAAGTCAGCTTG
AKAP9	Forward	GGCGTCATTGATGGCTATGCAG
AKAP9	Reverse	GCTGTTGCTCTGCCTCCAATTC
FSD1	Forward	TCAAGACCAGGTTCACACAGCC
FSD1	Reverse	CTGGTAGCACTGCCTCGCTTTT
WDR8	Forward	GGCACTGCAAAATAGACGAGGG
WDR8	Reverse	CACAAGGACCAGACGGTTATCC
SSX2IP	Forward	GACTTTTGCCAGACACGTTCCTG
SSX2IP	Reverse	AGGTCTTGACGCCACACTCCAT
CLIP170	Forward	AGAAGACGCTGCTGGACACAGA
CLIP170	Reverse	TGGCATCTTCCGCTGTTTGAGC
RAC1	Forward	CGGTGAATCTGGGCTTATGGGA
RAC1	Reverse	GGAGGTTATATCCTTACCGTACG
CDC42	Forward	TGACAGATTACGACCGCTGAGTT
CDC42	Reverse	GGAGTCTTTGGACAGTGGTGAG
IQGAP1	Forward	CCGTGGATACTTAGTTCGACAGG
IQGAP1	Reverse	AGCGCAGGTAAGCTAACCGATC
TUBG1	Forward	CACTCAAGAGGCTGACGCAGAA

TUBG1	Reverse	GGTTGATCTGGGAGAAGGATGG
TUBG2	Forward	TACCTCCTGGAGCGACTGAATG
TUBG2	Reverse	GCTGAACCACTACGTCGCTCAT
TUBGCP2	Forward	TACTCCGCTGTGACCAGGTTCA
TUBGCP2	Reverse	CCAGAATCAGGTGCTCCTTCAC
TUBGCP3	Forward	TAGTGGACCACTGCCAAGGAAG
TUBGCP3	Reverse	GATGAGACACGAGGCTGAGGAT
TUBGCP4	Forward	CTTGGTGGACTTTGAACAGGTGG
TUBGCP4	Reverse	GCCTGAAACAGTTCTCCACGTC
TUBGCP5	Forward	GATGCAGTCCATTGCTGAAAGCC
TUBGCP5	Reverse	CTCGTGAAAGTCACTCTGCTCC
TUBGCP6	Forward	GGTGTTCAGAGACGCTTATGGC
TUBGCP6	Reverse	CCACCTCTTTGGAGATGAGCAC
MATCAP	Forward	GCCATATTGCCAGCAAGAGC
MATCAP	Reverse	ATCGATGGTCTGGCGATGTC
NEDD1	Forward	GCTCTTTGTAACCATAGGCTTGG
NEDD1	Reverse	CCAAAGTGGCTCCATCAGGCAT
PCM1	Forward	CTCTACACCCAGTGAACAGCAG
PCM1	Reverse	CTGCTTTCCACCTCCTCTTCAG
CETN2	Forward	CCAAGGAGTTGGGTGAGAACCT
CETN2	Reverse	GCAGGAACTCTTGCTCACTGAC
CAP350	Forward	CAAAGCAGCTCAGGTCCATGCA
CAP350	Reverse	TCTGTGGTGAGGCGAGCAGTTT
CEP152	Forward	GAGCCTTGGAAGAACTTCGTGG
CEP152	Reverse	TGCAGCTCGTTCCTTACTCTCC
CEP192	Forward	ACCGTCACTCTCACTGCCATTG
CEP192	Reverse	AGGGCTTTCCAGCCTCCATAAG
CEP120	Forward	GAGAGAAGGCATAGACTCCCAG
CEP120	Reverse	GACTGGTTGTGAGATGGAACAGG
EB1	Forward	TGTGGCTGCCAGACAAGGTCAA
EB1	Reverse	GCAGCGGTTCTCTGTGTTGAGA
NUMA1	Forward	GGTTCCAGGAAGAGAGGCAGAA
NUMA1	Reverse	CTTGCTGGCTTGGTCAGAGTCA
KIF3A	Forward	GTTTGGACTATGCTGATGGCTGC
KIF3A	Reverse	GTTGCCGAATGTTCTCCAGTAGG
DCTN1	Forward	CAATGTGGAGCCTCTCACCAAG
DCTN1	Reverse	TCCTACCTCCACACTCATGCAG
LIS1	Forward	GTACTGGCATGTGCCTTATGACC
LIS1	Reverse	CGTAGGGTCTTGTCATCAGCAC
NUDEL	Forward	GGCACAAAGTTCTCTCGATCAGG
NUDEL	Reverse	CTCACACACTGAGAGGCAGCAT
EGFR	Forward	AACACCCTGGTCTGGAAGTACG
EGFR	Reverse	TCGTTGGACAGCCTTCAAGACC

beta Actin	Forward	CACCATTGGCAATGAGCGGTTC
beta Actin	Reverse	AGGTCTTTGCGGATGTCCACGT

Supplementary Table S2. siRNA sequences used in our research.

**siRNA**	**Sequences (5'-3')**
siCAMSAP3-1	CCGCCACCAUCCACUGCUA
siCAMSAP3-2	GCUGAUGGACGACCUCGAUAA

Supplementary Table S3. shRNA sequences used in our research.

**shRNA**	**Sequences (5'-3')**
shControl	GCAGTTATCTGGAAGATCAGG
shCAMSAP3	GCTGATGGACGACCTCGATAA

Supplementary Table S4. Primer for plasmids sequences used in our research.

Plasmid	**Sequences (5'-3')**	
FLAG-CAMSAP3-FL	FLAG_*NheI*_ Forward	CTAGCTAGCATGGATTACAAGGATGACGACGATAAGgtggaggcggcgccc
	*NOTI*_ Reverse	ATTTGCGGCCGCtcatttgggggtgccgcc
FLAG-CAMSAP3-△CH	FLAG_*NheI*_ Forward	CTAGCTAGCATGGATTACAAGGATGACGACGATAAGtttgcagaacgcaaga
	*NOTI*_ Reverse	ATTTGCGGCCGCtcatttgggggtgccgcc
FLAG-CAMSAP3-△HCKK	FLAG_*NheI*_ Forward	CTAGCTAGCATGGATTACAAGGATGACGACGATAAGgtggaggcggcgccc
	*NOTI*_ Reverse	ATTTGCGGCCGCtcactccagcaggctggcc
FLAG-CAMSAP3-HCKK	FLAG_*NheI*_ Forward	CTAGCTAGCATGGATTACAAGGATGACGACGATAAGcggcagcagcggcga
	*NOTI*_ Reverse	ATTTGCGGCCGCtcatttgggggtgccgcc
