# Supplementary figures and images for: Cytoskeletal remodeling via CAMSAP3 downregulation drives resistance to osimertinib in NSCLC cells
Source: Cell Death Dis. 2025 Dec 11;17(1):90. doi: 10.1038/s41419-025-08299-0 (PMC12830379; doi:10.1038/s41419-025-08299-0)

Fig2B:

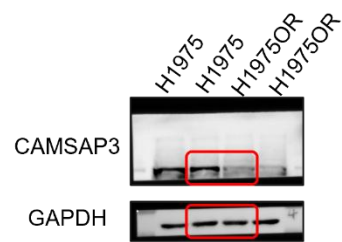

Fig3A:

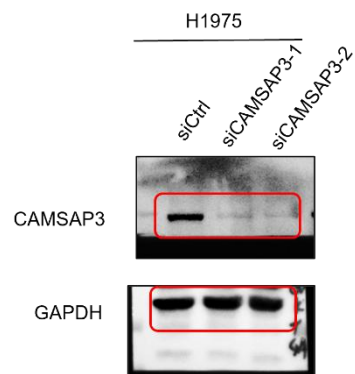

Fig4B

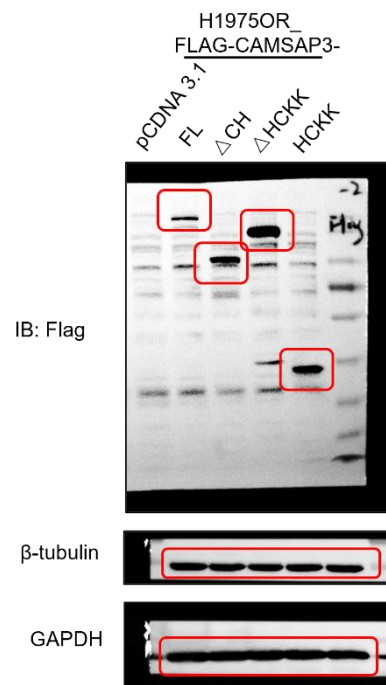

Fig5B:

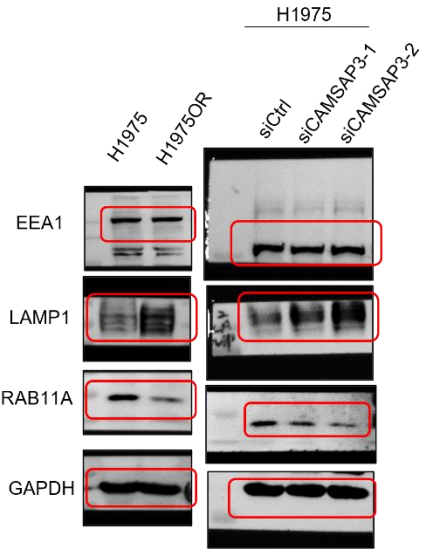

Fig5E:

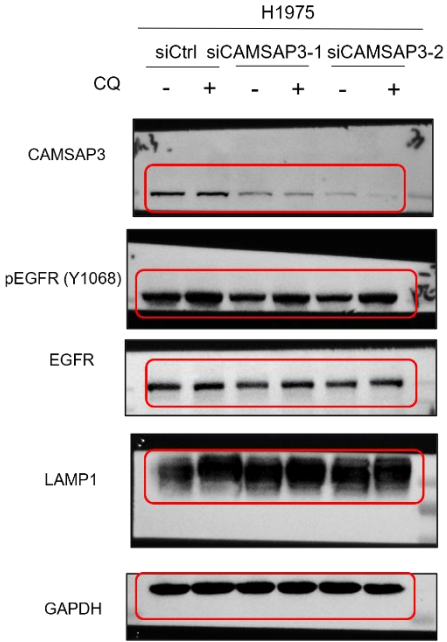

Fig6B:

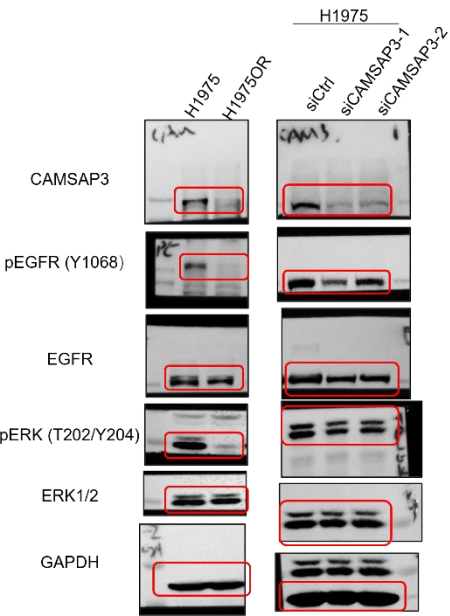

FigS2B:

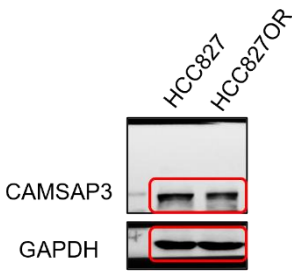

FigS3A:

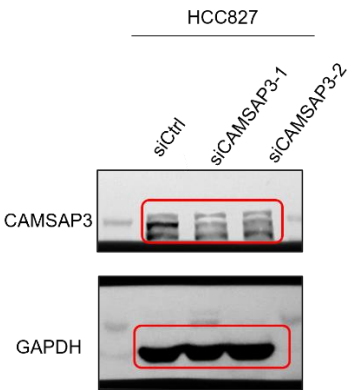

FigS3E:

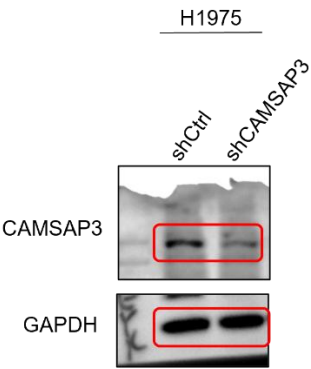

FigS4B:

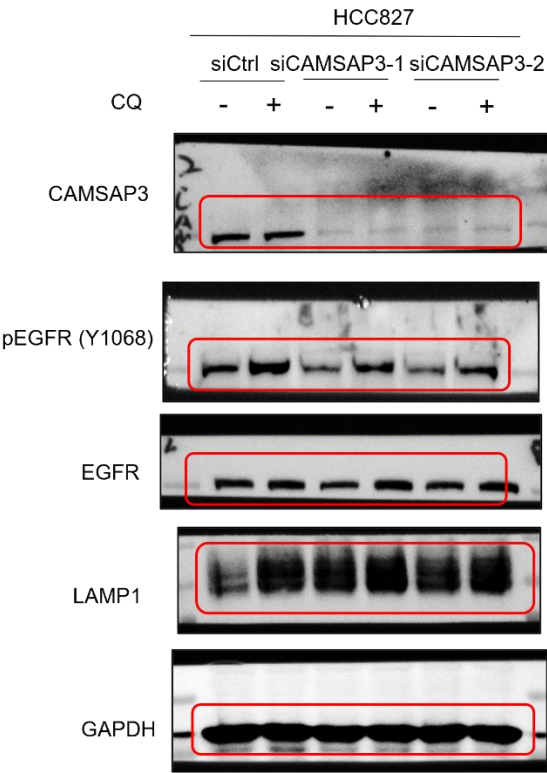

FigS5B:

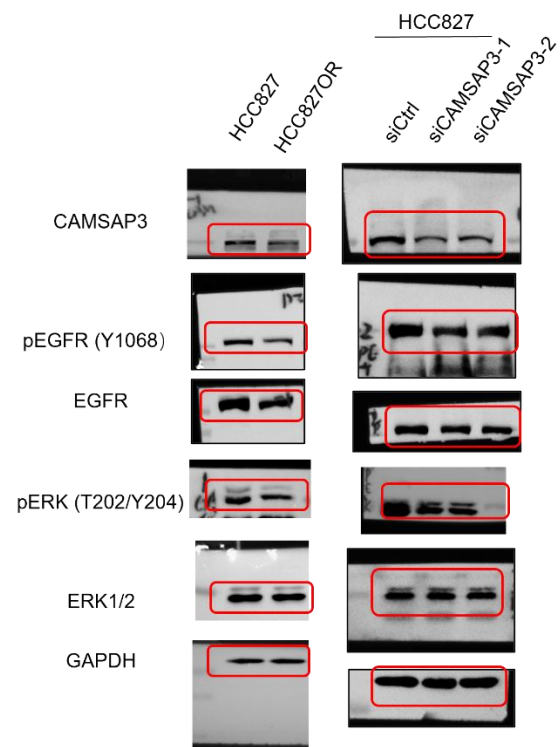

Supplement: Supplementary file 2 — Original western blots [file 41419_2025_8299_MOESM2_ESM.pdf]
